# Proinsulin is stable at room temperature for 24 hours in EDTA: A clinical laboratory analysis (adAPT 3)

**DOI:** 10.1371/journal.pone.0171716

**Published:** 2017-04-20

**Authors:** Jane Davidson, Timothy McDonald, Calum Sutherland, Mohammod Mostazir, Lidy VanAalten, Terence Wilkin

**Affiliations:** 1 Cardiovascular and Diabetes Medicine, University of Dundee, Dundee, United Kingdom; 2 NIHR Clinical Research Facility, Blood Sciences, Royal Devon and Exeter NHS Foundation Trust, Exeter, United Kingdom; 3 Institute of Health Research, University of Exeter Medical School, Exeter, United Kingdom; 4 College of Life and Environmental Sciences (CLES), University of Exeter, Exeter, United Kingdom; Helsingin Yliopisto, FINLAND

## Abstract

**Aims:**

Reference laboratories advise immediate separation and freezing of samples for the assay of proinsulin, which limit its practicability for smaller centres. Following the demonstration that insulin and C-peptide are stable in EDTA at room temperature for at least 24hours, we undertook simple stability studies to establish whether the same might apply to proinsulin.

**Methods:**

Venous blood samples were drawn from six adult women, some fasting, some not, aliquoted and assayed immediately and after storage at either 4°C or ambient temperature for periods from 2h to 24h.

**Results:**

There was no significant variation or difference with storage time or storage condition in either individual or group analysis.

**Conclusion:**

Proinsulin appears to be stable at room temperature in EDTA for at least 24h. Immediate separation and storage on ice of samples for proinsulin assay is not necessary, which will simplify sample transport, particularly for multicentre trials.

## Introduction

Proinsulin is the prohormone of insulin, and is packaged in the beta cell by a ‘clip’ or connecting C-peptide. The clip is shed by protease cleavage as the intact proinsulin molecule is secreted, releasing equimolar quantities of C-peptide and active insulin into the circulation [1]. In health, the majority of proinsulin is processed in this way, so that only small amounts of intact proinsulin escape from the beta cell.

However, when stressed by the increased demand for insulin imposed by insulin resistance, beta cells become progressively less able to cleave proinsulin,[2] as a result of which the circulating concentration of intact proinsulin and the proinsulin:insulin ratio rises. Insulin resistance is believed to underpin type 2 diabetes, and intact proinsulin has become an important marker of metabolically stressed beta cells in both diabetes and prediabetes.[3]

Reference laboratories in the UK advise plain plastic (serum) or lithium heparin bottles (plasma) for the collection of samples for proinsulin, with immediate freezing after separation and transport on dry ice.[4] However, the same advice applied to the collection of samples for insulin and C-peptide measurement, until a recent report in this journal from this laboratory showed that both were stable, unseparated and at room temperature, for at least 24 hours–provided they were preserved in EDTA (ethylene-diamine-tetra-acetic acid) and not lithium heparin.[5] We wondered whether the same might apply to intact proinsulin, as it would simplify sample transport considerably. Accordingly, we collected serum aliquots into EDTA bottles, and measured proinsulin on several occasions over 24 hours to assess deterioration of the sample over time.

## Samples and methods

### Samples

Six healthy non-diabetic volunteers (all female), median age 36 years (range 27–53 years), mean BMI 26.4 kg/m2 (range 22.5–31.3) provided samples for the study. Two of the subjects were fasted, and the others post prandial, at the time of blood sampling.

The samples were excess clinical samples, anonymised before analysis, and used ethically in line with the UK Human Tissue Authority’s directive on ‘Use of residual clinical material for evaluation and assessment of in vitro diagnostic performance’ (Human Tissue Authority, code of practice 9).[6] As an analysis of sample stability, the study was categorised as assay performance analysis rather than clinical research.

### Stability studies

Venous blood was collected from an ante-cubital vein of each of the volunteers and aliquoted into twelve 3ml K^+^ EDTA BD Vacutainer tubes (BD Biosciences, Oxford, UK). Tubes were inverted 10 times. Two of the aliquots were centrifuged immediately, while five were left as whole blood to stand at room temperature (RT) and five at 4°C. At each subsequent time point (2, 6, 8, 12 and 24 hours), a further aliquot from each storage condition was centrifuged at 1300xg for 15 minutes at RT in a Beckman Coulter Allegra X-12 centrifuge (note tubes stored at 4°C were placed at RT 30 minutes before spin). From each, 160μl of plasma (upper layer) were placed into a 3.5ml graduated storage vial (Elkay Labs, Basingstoke, UK) and stored at -80°C before being shipped frozen on dry ice to Exeter for batched analysis.

### Analyses

Intact proinsulin was assayed using a commercial colorimetric two-site sandwich ELISA (Teco, Switzerland) on a Dynex DS2 automated ELISA reader (Launch Diagnostics, Longfield, UK) giving an intra assay CV <2.4%, inter assay CV < 4% and a limit of detection of 0.3 pmol/L. The intact proinsulin assay is highly specific and has no cross reactivity with insulin, C-peptide or either split and des forms of proinsulin. Proinsulin estimates in three of the 36 samples analysed in this study lay below the limit of detection, and were reported as 0.3 pmol/l. Plasma insulin was measured using an electrochemiluminescence immunoassay on a Roche E170 analyser (Roche Diagnostics, Mannheim, Germany) which has an intra assay CV <3.2%, inter assay CV < 4.1% and a limit of detection of 3.0 pmol/L. The assay employed two mouse monoclonal anti-insulin antibodies, the first labelled with acridinium ester, and the second covalently coupled to paramagnetic particles. Both analyses were carried out at the Clinical Chemistry Department of the Royal Devon and Exeter NHS Foundation Trust, Exeter, UK.

### Statistical presentation

Arithmetic means/±SDs are presented for proinsulin, insulin and proinsulin-insulin ratio at each time point at both 4°C and at room temperature. Subject-specific measures are plotted for each variable under both conditions. Since individuals were measured repeatedly, a separate linear mixed effect random intercept model was fitted to test whether the time-related linear trend at 4°C was different than the trend at room temperature. The log-likelihood ratio test statistic for model comparison showed significantly better fit in favour of random intercept models compared to the single level regression model for all three outcomes (Proinsulin: X^2^ 210.60, p<0.001; Insulin: X^2^ 229.04, p<0.001; Proinsulin-insulin ratio: X^2^ 230.69, p<0.001). All outcomes were log transformed before fitting the model, and expected means/95% CI were plotted after back transformation. Overall, 69 observations contributed to the model for proinsulin, 66 for insulin and 63 for proinsulin/insulin ratio.

## Results

**Fig 1:**
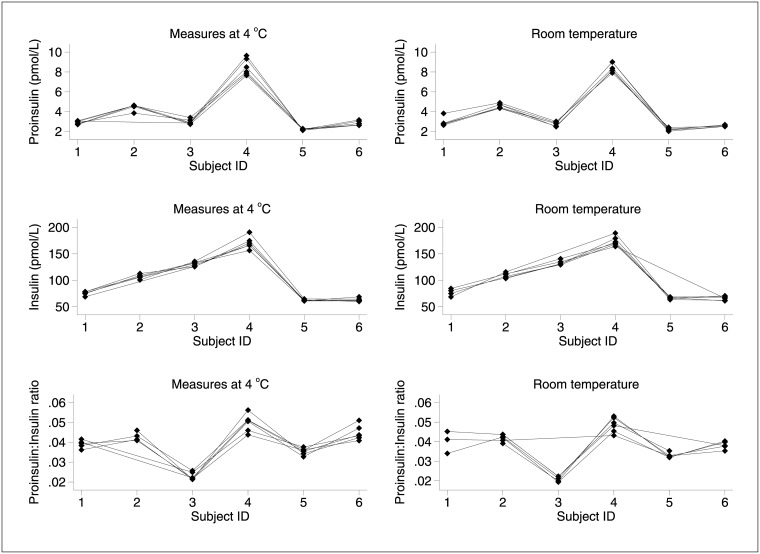
Individual data–within-subject variation in measures of proinsulin (upper panel), insulin (middle panel) and proinsulin:insulin ratio (lower panel) at collection (0h) and at the five time points thereafter, in samples kept at 4°C (left-hand panels) and at room temperature (right-hand panels) for the duration.

The range of proinsulin tested was 0.3–9.6 pmol/L, and that of insulin 60–190 pmol/L. Within-subject variation (Fig 1) was small for all measures, suggesting that proinsulin and insulin were stable in EDTA over time, whether at 4°C or at room temperature. There were no significant differences in the means and variance of the two conditions.

**Fig 2:**
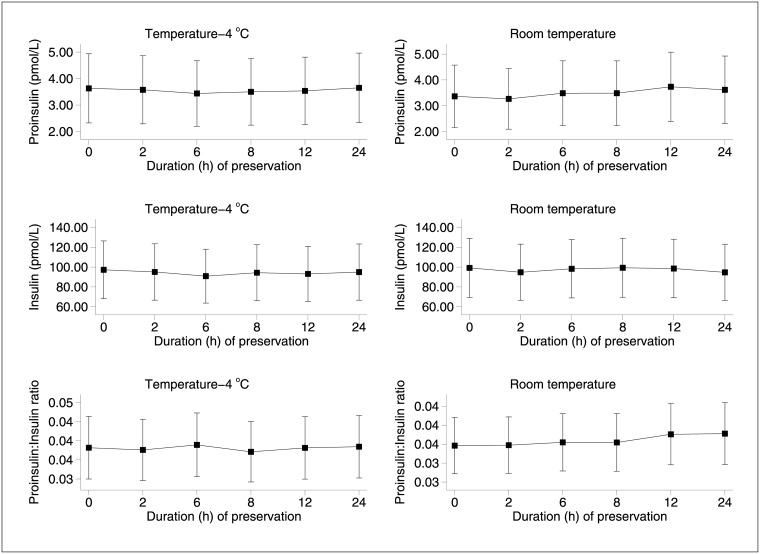
Group data–between-subject variation (mean concentrations, 95% confidence limits) of intact proinsulin (upper panel), insulin (middle panel) and the proinsulin:insulin ratio (lower panel) at collection (0h) and at the five time points thereafter, in samples kept at 4°C (left-hand panels) and at room temperature (right-hand panels) for the duration.

The group data in Fig 2 show the estimated means (back transformed) for all three outcomes, and confirm how both intact proinsulin and insulin remained stable in whole blood at 4°C and at room temperature for at least 24 hours in EDTA, and possibly for longer. The mixed models showed no difference in trend over time when comparing the two conditions, and the X^2^ tests of overall trends (time x condition interaction) were not statistically significant at p = 0.08 (proinsulin), p = 0.12 (insulin) and p = 0.57 (proinsulin to insulin ratio).

## Discussion

Reference laboratories and assay kit manufacturers advise that blood samples for the measurement of intact proinsulin be processed immediately, and frozen for transport. This study reported here suggests that, provided EDTA is used as the vehicle, proinsulin can be stored for at least 24 hours at room temperature without the need for separation.

This laboratory showed previously that the same is true for insulin and C-peptide in EDTA.^5^ Sample collection for proinsulin, like that of insulin, tends to be confined to larger centres, not because of cost, but because of the logistics of centrifuging and freezing multiple samples peripherally, and of putting them on dry ice for transport centrally. Our data indicate that it is possible to store/transport whole blood in EDTA at room temperature for at least 24 hours, without fear of deterioration of intact proinsulin or insulin. The findings may have particular significance for multicentre trials, where smaller study sites lack the provision or personnel for sample separation/storage/specialised transport. Raw blood samples can now be handed to a courier for same day transport to the study lab where separation, triage and labelling can all be carried out centrally without risk that the sample will spoil.

## Conclusion

Pre-diabetes is increasingly recognised as a disease state, and proinsulin or the proinsulin:insulin and proinsulin:C-peptide ratios as markers for it. Reference laboratories recommend immediate separation and freezing of proinsulin samples, which makes their preparation, storage and transport problematic. This report shows that proinsulin (as demonstrated recently for insulin and C-peptide^5^) can be stored/transported unseparated at room temperature for at least 24 hours in EDTA. Proinsulin can be dispatched as whole blood in EDTA without preparation, which will make it more available for both assay individuals and multicentre trials.
